# Pathology and Treatment of Yellow Fever; with Some Remarks upon the Nature of Its Cause and Its Prevention

**Published:** 1881-08

**Authors:** H. D. Schmidt

**Affiliations:** New Orleans, La.


### ARTICLE IV.

### THE PATHOLOGY AND TREATMENT OF YELLOW FEVER; WITH SOME REMARKS UPON THE NATURE OF ITS CAUSE AND ITS PREVENTION. By H. D. Schmidt, M.D., New Orleans, La.

(Continued from page 70, July No., 1881.)

### GENERAL PATHOLOGY.

We have now studied the clinical phenomena of yellow fever, together with the pathological changes taking place in various tissues and organs during the course of the disease, and revealed by a post mortem macroscopical and microscopical examination; a comparison of these two sets of phenomena with each other, for the purpose of determining, as far as possible, the relationship necessarily existing between them must, therefore, be the next object in view. Formerly, when diseases were classified by certain groups of clinical phenomena, or symptoms, which they presented, it was chiefly these symptoms to which the physician directed his attention, and by which he was guided in his treatment, consisting for the most part of remedies to which, in the cure of each particular disease, a specific action was attributed. With the rapid development of pathological science, however, the custom of treating disease in this empirical way was gradually abandoned for more rational methods, based upon a thorough knowledge of the intrinsic nature of the disease, obtained from the nature of the pathological changes observed after death in the tissues and organs. But even a knowledge of these changes, wrought upon the internal organs by the morbid process of one or the other disease, is insufficient to safely guide the physician in his efforts to obtain a cure, unless he is able to tell from the symptoms, presented by the particular condition of his patient, the exact and simultaneous condition of these organs; in other words, to form an accurate scientific diagnosis, the highest accomplishment of the practicing physician. In a considerable number of diseases, especially those of the heart and respiratory organs, medical science has reached this aim, and I am confident that, to a considerable extent, the same may also be accomplished in yellow fever.

In yellow fever, as in other acute infectious diseases, the most prominent phenomenon observed is that of fever; I shall, therefore, introduce my remarks on the general pathology of this disease with a brief consideration of the observed facts, relating to the *febrile process in general.*

Various theories on the phenomenon of fever have been advanced by medical men at all periods of medical history. The older theories were all based upon the principles of "humoral pathology," according to which not only fevers, but all other diseases depended upon a contamination of the blood, caused by the reception of noxious substances from without, or, in consequence of certain changes going on within the body, the febrile process being an effort of nature to expel the noxious matter. It was *Hoffmann*,* who first directed the attention from the fluids to the solid tissues, as the seat of the disease. He sought the source of fever in the nervous system, believing that it was orig-

* *G. B. Wood*, "Practice of Medicine," fourth edition, Vol. I, p. 101.

inally depending upon a spasm of the capillaries, and considering the heat of the skin and the arterial excitement which follow as the mere reaction of the system, necessary to overcome this spasm. A very similar theory was entertained and promulgated by *Cullen*, and, also, in a more or less modified form, by some other authors. Another theory of fever was, in the beginning of this century, put forth by *Clutterbuck*,* who regarded all forms as of local origin, and depending upon local inflammation. The so-called idiopathic fevers, therefore, he regarded as inflammation of the brain, designating them "encephalitis." *Broussais*, in announcing his doctrine of fever, agreed with Clutterbuck in denying the existence of essential fevers, but differed from him in ascribing all those forms, previously denominated idiopathic, to inflammation of the mucous membrane of the stomach, or that conjointly of the stomach and bowels; in other words, being *gastritis* or *gastro-enteritis*. The impression which Broussais's theory had made, gradually faded, and there has been no doctrine of fever since which had a general prevalence, though the above theories of fever have, with various modifications, extended into our time. Thus, the neuro-pathological theory of Hoffmann and Cullen was, in a modified form, subsequently more developed by *Virchow*,† who regarded the phenomena of the febrile process, especially the elevation of temperature, as depending upon a depression (Nachlass) of certain parts of the nervous system, which he supposed to act as moderators of the production of heat. In still more recent times, however, the old theories based upon humoral pathology, appear to recover their lost grounds.

Although the most prominent symptom of fever, the abnormally increased heat of the body, has always been attributed to an increased waste of matter derived from the tissues, the discovery of a correspondingly increased formation of urea, found in the urine during the febrile process, and verifying this supposition, belongs to more recent times. The precise nature of the febrile process itself, and the true cause of the increase of bodily temperature, however, remained still unknown, and gave rise to

* L. c., page 103.

† *R. Virchow*.—Handbuch der Pathologie u. Therapie, 1854, Vol. I, p. 33.

very numerous and laborious investigations, made by a considerable number of investigators, such as *Jochmann*, *Traube*, *Moos*, *Redenbacher*, *Uhle*, *Ringer*, *Brattler*, *Wingl*, *Wachsmuth*, *Warnecke*, *Huppert*, *Tschsschichim*, *Breuer*, *Chroback*, *Leyden*, *Ranke*, *Senator*, *Naunyn*, *Unruh*, and others.* A most able treatise, covering the whole subject, and embracing his own labors as well as those of others, was written by *Senator*, who, for his extended contributions to the knowledge of the febrile process, as far as it goes, may be regarded as a prominent authority. And as some of his special views seem to have been corroborated by still more recent observations, I shall not hesitate to extract from his treatise in the following discussion.

In the above mentioned investigations, the attention was particularly directed to the existing proportion between the production and discharge of heat, carbonic acid and water; and the most prominent facts thus far elicited were: an increased formation of urea in the urine, and an augmentation in the discharge of carbonic acid, water and heat.

The increased formation of urea is not depending upon a greater activity of the normal exchanges of matter in general, but rather upon a greater waste of albumen. The quantity of urea excreted during the whole fever amounts in the average to more than twice the quantity excreted during the absence of fever, but otherwise under the same conditions; that is, when the body is kept on fever diet. The average quantity of urea excreted on such diet, and taken by Senator as a basis, was 18 grms. in twenty-four hours.

The increase in the quantity of urea secreted begins, as was first observed by Ringer, Traube, Joachmann, Uhle and Redenbacher, before an elevation of the bodily temperature is noticed. Senator, who corroborated this fact by his experiments on dogs, in which, contrary to man, the activity of the urinary function increases during the febrile process, enlarges upon this phenomenon as follows: "Whilst, namely, with the dog the quantity of

---

* *Virchow u. Hirsch*, Jahresberichte, etc., für die Jahre 1866–1872. Section: Allgemeine Pathologie.

† *Senator*.—"Untersuchungen ueber den Fieberhaften Process, und seine Behandlung.' Berlin, 1873.

urea discharged with the urine in about twenty-four hours may be looked upon as equal to that *formed* in the same time, such a presumption is not accepted as regards most febrile diseases in man. In the dog, at least in artificially produced pyæmia, the discharge of urea is favored by the augmentation of the water of the urine; and a loss of nitrogen by some other way than through the urine, as, for example, through the fæces, did not take place during the time of observation. With man it is different. Here, the amount of urine is usually diminished, and with this the chief source for the abstraction of nitrogen is limited. In consequence, the removal of the latter does not keep equal pace with the extensive disintegration of albumen; urea—or, as we may for the present suppose, bodies formed preliminary to urea—accumulate in the body, to be afterwards eliminated at a shorter or longer time after the defervescence of temperature, as the post-febrile augmentation of the discharge of the urea so frequently shows. Besides this, the last residue of oxydized nitrogen is certainly as yet not removed with the latter, but even in the succeeding days the origin of a portion of the discharged urea may still be referred to the time of fever. Moreover, the post-febrile augmentation of urea does not always show itself very conspicuously, as, quite frequently, the body rids itself of its surplus only very gradually, and an imperceptible transition from the febrile to the normal excretion of urea takes place, so that it is difficult to determine how far this is still depending upon the influence of the fever." He further remarks that in different febrile diseases, much nitrogenous material, in various other forms than the ordinary constituents of urine, leaves the body, or, at least, is abstracted from the exchanges of matter, not only with the stools, but with the expectoration, or in the form of albumen in the urine, as it frequently occurs with high temperatures. These losses, which affect the organism only during the fever, but not when in a state of inanition, also bear in favor of a febrile augmentation of the exchange of albumen; for, if these quantities of nitrogen were not abstracted from the exchange of matter, the organism would discharge still more urea during the fever. According to *Hoppe*, the quantity of ammonia in the urine, also, is considerably augmented in febrile diseases forming an expenditure of nitrogen not taken as yet into account in determining the quantity of urea. If, therefore, in the fever of the dog, the augmentation of the excretion of urea may be regarded as in just proportion to that of the disintegration of albumen, this is not applicable in the same degree to man. On the contrary, the disintegration of albumen during the fever of man is augmented in a greater measure, as can be calculated from the increase of the urea, and must, therefore, amount to more than double the exchange taking place under the same conditions without fever.

The conditions for the discharge of carbonic acid are, according to Senator, during the hot stage of the fever better than normal; under the most favorable circumstances, the discharge during the day-time is augmented from thirty to forty per cent., while during the night, it is probably, as in the normal condition, generally less. The formation of carbonic acid during fever, therefore, can, in the most favorable case, only augment to below thirty to forty per cent.

From the above facts, Senator concludes that in the fever of man no uniform augmentation of the whole exchange of matter, viz., the albumen and fat, takes place. As regards the disintegration of the constituents of the body during the febrile process, however, there are other facts known, which not only confirm it, but moreover indicate, which particular tissues are affected by the augmented disintegration. Above all, it is the exact investigations of *Salkowski* upon the excretion of the alkaline salts, which have shown that the quantity of potassa excreted by a healthy individual on fever diet, is from three to four, or even seven, times augmented during the febrile process, while a similar augmentation of soda does not take place. The latter, on the contrary, appears to be excreted in a smaller quantity, which is perhaps owing to the existing antagonism in the excretion of potassa and soda, shown by *Boecker* and *Reinson*, and more recently by *Bunge*. It is furthermore known that in febrile diseases the coloring matter of the urine considerably augments in quantity, which, according to *Vogel*, amounts to even more than four times the quantity excreted by healthy individuals on an ordinary full diet. No final product is augmented in the same measure as the urea, coloring matter of the urine, and the potassa, not even the phosphoric acid; it is even doubtful, after the careful determinations made by *Rosenstein*, whether its excretion—in the urine—is much augmented under the influence of fever. As regards the excretion of sulphuric acid, the greater part of which is likewise derived from the combustion of albumen during the fever, nothing certain is known, probably because its secretion does not regularly and rapidly follow upon its formation.

Those nitrogenous tissues which are rich in potassa and hæmoglobin, the mother substance of the greater part of urea, are therefore, especially prone to disintegration, namely: The colored blood corpuscles first of all, and then the muscles, while the brain and spinal marrow, with the nervous tissues in general, though likewise rich in potassa, may be excluded, as they furnish no coloring matter of the urine, and also because the discharge of phosphoric acid is not augmented. Experience teaches besides, that the central parts of the nervous system are least affected by the febrile consumption, or by that caused by inanition. The fact, that it is the colored blood corpuscles which, first of all, suffer and disintegrate during the febrile process, is corroborated by the observations of *Koerber*, which showed that the decomposability of the hæmoglobin was increased in febrile diseases, and the statement of *Mannasseïn*, concerning the general diminution of the colored blood corpuscles in size during fever and with an elevated temperature. But if those corpuscles and the hæmoglobin are destroyed to a great extent; if, in consequence, the body becomes poorer in those elements which effect the reception of the oxygen from the air and its conveyance to the tissues, and if other elements do not take charge of this work, then the activity of the processes of combustion in the body must necessarily decrease in the same measure for want of oxygen. The further statement of Mannasseïn, that blood corpuscles rich in oxygen, increase in size, as also when under the influence of certain anti-febrile remedies—quinine, etc.—is a further proof that the function of the colored blood corpuscles is lowered during the fever; that the latter appropriate less oxygen, rapidly decrease in size and disintegrate.

Accordingly, the organism cannot absorb as much oxygen, and in consequence cannot oxydize as much material of the body to its final products during fever, as it can without fever and under the same nutritive conditions.

If, now, the one final product, urea, is found in too large a quantity, already consuming a certain quantity of oxygen more than usual, it follows that the other final products, which require still more oxygen, must naturally be diminished in quantity; and from this, it again follows that the combustion of the non-nitrogenous constituents of the body—the fat—and, accordingly, also the formation of carbonic acid during fever, cannot be simply augmented. The increased destruction of the colored blood corpuscles, the carriers of oxygen, then, forms another cause for the granular and fatty disintegration of the tissues, taking place here in a manner similar to the effect of poisons which act by abstracting the oxygen. The conclusion, therefore, is that in the majority of febrile diseases of man, the body decomposes exclusively, or, at least, more than normally,nitrogenous material, *i. e.*, albumen, and in consequence becomes relatively richer in non-nitrogenous, *i. e.*, fat. Without oxydation, only water, increased in quantity, can be formed from the ultimate products of the exchange of matter—by synthesis and dishydration.

The quantity of urine in fever is usually in proportion to the supply of liquids, but its secretion is more unfavorable than in the normal condition, as a comparatively smaller portion of the received water is discharged by the urine than in the absence of fever, but otherwise under the same conditions. The quantity of the evaporated water is augmented during the fever, and amounts to even a little more than that of the expired carbonic acid; therefore, the quantity of water lost by evaporation is comparatively large. In what proportion the whole loss of water stands to its formation during the fever—with the exception of extraordinary losses—can as yet not be determined. With a large supply of water some of it may, as in the normal condition, be retained. The variations in the weight of the body during fever chiefly depend upon these unstable relations between the supply and loss of water.

In comparing the economy or exchange of matter during the febrile process, as above stated, with that taking place in an individual kept on a perfectly normal diet, Senator remarks that the only two substances of excretion, from which the exchange of of albumen and fat may be calculated, are urea and carbonic acid, and that the daily normal excretion of the former amounts from 25 to 30 grms., while that of the latter is from 700 to 800 grms. This, based upon the values of combustion as determined by *Frankland*—4,263 units of heat for 1 grm. albumen, and 9.1 units for 1 grm. fat—corresponds to an exchange of 74 to 93 grms. albumen and 193 to 240 grms. fat, with a formation of heat of 2072 to 2580 units. The fever patients upon whom the investigations relating to the excretion of urea and carbonic acid were made, rather belonged to those who in their ordinary normal condition show a low equivalent weight of nitrogen, and whose daily excretion of urea deviates perhaps little from 25 grms. If these individuals discharge, at least during the first days and with a high fever, 40 to 50 grms. in 24 hours, then this is, in the most favorable case, a surplus expenditure of 25 grms. at the utmost in comparison with their normal condition, or a surplus exchange of 74 grms. albumen at the highest rate. In supposing that this albumen were completely oxydized, as in the healthy condition, to form urea, $C O^2$ and $H O^2$, an augmentation of heat, equivalent to 315 units, would result, or more still, because during the fever more albumen is disintegrated than corresponds to the urea.

In corroboration of the statements and views of Senator, but more especially for their own intrinsic value, I will recite some of the more recent investigations regarding the exchange of matter during fever and inanition, obtained by *Zuelzer*.* They are as follows: "1. The total sum of the nitrogenous excretions is augmented during the state of febrile excitement, and diminished during the state of depression—inanition, convalescence. 2. The relative quantities of phosphoric and sulphuric acid—in proportion to the nitrogen—in the urine during the state of fever and hunger—do not exceed those found in muscle and brain. 3. In proportion to the nitrogen, more sulphuric acid is excreted in the state of fever and inanition, and less during convalescence than is excreted with a meat diet. Accordingly, these two final products, arising from the disintegration of the albuminous sub-

**Virchow u. Hirsch*, Jahresbericht fuer das Jahr, 18 Vol. I., p. 220.

stances, are excreted through the urine in a relatively larger quantity than in the normal condition. During convalescence, however, the urine also contains—in a larger quantity than during inanition—nitrogenous substances, not derived from disintegrated flesh, but from the nervous substance, that is, from a tissue, in which the nitrogen is not associated with sulphur. Besides, the albuminous substances supplied for the reconstruction of the wasted constituents of the body, are mostly used in the body itself, while the secretion of bile is simultaneously augmented. 4. The relative quantity of the phosphoric acid is less during the fever than with a meat diet; during convalescence and inanition, however, it is larger, though smaller than on feeding with brain. During the fever, therefore, and simultaneous with the augmented disintegration of the nitrogenous tissues, phosphoric acid is retained in the organism to be excreted after the disappearance of the fever. In inanition—together with the diminished disintegration of the nitrogenous constituents of the body—the relative quantity of phosphoric acid in the urine is augmented. 5. Accordingly, the exchange of muscular substance, is augmented during the fever, whilst in inanition, as in convalescence, the exchange affects more the nervous tissue, which, in virtue of its richness in lecithin and cephalin (Thudichum) furnishes comparatively less nitrogen, but more phosphoric acid. 6. These processes in the nervous substance cannot be referred to a simple increase or decrease, as, during the state of inanition, according to the law of *Voit* concerning the loss of tissue during the state of hunger, the total mass of nervous substance experiences the least loss. The regular change from retention to augmented secretion of the phosphates under existing and depressing influences, as also the widely varying results of the elementary analysis of the brain, rather indicate that the quality of the constituents of the tissues may experience rapid and intense changes."

The elevated temperature of the body in fever has usually been attributed to the increased disintegration or waste of tissue taking place during that process and liberating a surplus quantity of heat. The more recent investigations, cited above, however, have shown that the heat developed from this surplus exchange of matter is not sufficient to account for that discharged from the surface of the patient, and that there must be other sources besides. The investigations of Leyden and Senator, particularly, have furthermore demonstrated that the discharge of heat during the whole course of the fever, though augmented, is nevertheless subject to considerable variations. The quantity of heat stands in no fixed proportion with the temperature of those accessible parts of the body, upon which it is usually measured, but may be less with a high temperature than with a lower, and even sink to the normal standard; in the stage of defervesence, especially when a critical sweat occurs, the discharge of heat is highest, amounting to double and triple the normal quantity. During the hot stage, it is augmented to one and a half, or nearly double the normal amount.

In order to understand these variations existing between the formation and discharge of heat, we need only examine the particular mode in which the healthy organism regulates these processes in keeping up the equilibrium of heat. As the heat, derived from the exchange of matter constantly occurring in every tissue of the body, is distributed throughout the latter by the circulating blood, it follows that the richer any part or organ is in blood, the higher will be its temperature; but as the amount of blood which an organ contains is proportionate to the number and caliber of its minute blood-vessels, the arterioles, venules and capillaries, its temperature will change with the contraction or dilatation of these vessels. In the normal condition, the tonus of these vessels, especially that of the arterioles possessing muscular fibers, is kept up by nerves—vaso-motor—connected with certain nerve-centers, chiefly placed in the middle portion of the medulla oblongata, but also in the spinal marrow; it is these centers, therefore, that regulate the normal temperature of the body by the amount of contraction or relaxation which they impose upon the muscular fibers in the walls of the vessels, or, in other words, by the amount of blood which they suffer to pass through the organ. The whole apparatus is reflex in its nature, and set and kept in operation by the impressions made, particularly upon the external surface of the skin; and it is thus that the application of cold will contract the minute vessels of the latter, while moderate heat will cause them to become relaxed. To a certain extent, however, the discharge of heat from the body is also regulated by the lungs, the respirations increasing or diminishing in frequency; this is especially the case in animals clothed with a fur. Even in the normal condition, the quantity of heat produced within the body is not always the same, as, for instance, during the process of digestion, when by the more extended decombination and combination of matter taking place, a surplus of heat is produced; or, also, during muscular exercise, when more muscular substance is disintegrated than in a condition of quietude and repose. With man, the limits of this capacity of regulating the temperature in the interior of the body are quite narrow, lying between 27°–37° C. of the surrounding air; whatever is required beyond he has to replace by accessory means, as clothes, etc.

Now, as regards the discharge of heat during the febrile process, it is not augmented in the beginning or cold stage, but rather diminished; in the hot stage, or at the height of the fever, however, it is augmented 70–75 per cent. in the average during the day, and still more at the critical defervescence. As in the normal condition, the most heat is lost during the hot stage by conduction and radiation, while at the critical defervescence the discharge takes place by evaporation.

It has already been remarked that the augmented exchange of matter, indicated by the final products, urea and carbonic acid, does not account for the surplus of heat present in the body during the fever, and that there must be some other sources of heat besides. The most prominent of the latter are, according to Senator: 1. The consumption of those static forces, which, in the healthy organism, are stored up for the performance of any work. 2. The accumulation of heat during the pyrogenetic or cold stage of the fever. Besides these, other sources for the increased formation of heat may be sought in the greater disintegration and metamorphosis of albumen into urea, and in unknown processes concerning the formation of water.

With regard to the first of these sources, Senator presumes that the healthy body is in possession of latent forces, which are not generally liberated, but kept in store for extraordinary opportunities, such as an interrupted supply of nutriment, when they may be used, both for the performance of mechanical work, or for the development of heat. But as during the fever no external work of any import is done, these static forces are used up in the form of heat. Instead that in the healthy condition they are by the excitation of the will converted into mechanical work and heat, they will, on the other hand, under the influence of the febrile cause, be converted into heat only.

In the consumption of these static forces, which may either take place rapidly by mechanical work, or more slowly by hunger, carbonic acid is developed by the combustion of certain non-nitrogenous substances, containing these forces. The same should be the case, if the consumption of the latter takes place during the fever, to form a source for an augmented formation of carbonic acid during this process. When, nevertheless, such a source cannot be proved, or is not very conspicuous, even if present, no objection can be raised against the presumption that these static forces are liberated. For the surplus of carbonic acid formed here may be counterbalanced on the other side by being formed in a smaller quantity in such a manner that the albumen is not, as in the normal condition, completely consumed, but that it leaves behind, after the splitting off of the urea, a non-nitrogenous rest, more or less related to fat—the fatty degeneration of the parenchymæ in severe febrile diseases corroborates this hypothesis. The difference between the exchange of matter in a healthy and in a fevered individual is as follows: The healthy individual converts, under ordinary conditions, daily, a certain quantity of albumen and fat into the corresponding quantity of urea and carbonic acid, and replaces the loss by an equal quantity of fresh supply, keeping thus his store of static forces at the same level. If the fresh supply is insufficient, that is, during a more or less perfect state of hunger, then he consumes, in proportion to his state of nutrition, a quantity of albumen and fat of his body, and his store of static forces decreases. But the combustion is always perfect, that is, the quantity of albumen and fat destined to the exchange is completely converted into urea, carbonic acid and water. During work, he consumes the same quantity of albumen, with the only difference, that the static forces, which during the state of hunger are more gradually consumed, are here liberated in a short time, giving rise to a rapid development of carbonic acid, which otherwise would have been discharged very gradually. During the fever, a larger quantity of albumen is disintegrated as during inanition, but only to urea and fat. The static forces are, perhaps in consequence of the enormous destruction of albumen, more rapidly consumed, and, for this reason, more heat and carbonic acid result in an equal time from this consumption than in a state of hunger; the carbonic acid, however, which would have resulted from the complete combustion of the albumen, is left out, so that, on the whole, nothing, or only very little more than in the corresponding time of hunger, is formed.

As regards the accumulation of heat during the cold stage, it is presumed that it really occurs. Even the normal discharge of heat during this stage would result in a saving or accumulation of it, because the changes in the exchange of matter are already taking place previously to the commencement of the chill, and as conditions for an augmented formation of heat may be found, besides, in the phenomena accompanying the chill itself. This presumption is moreover corroborated by the commencing augmentation of the discharge of urea, and the already preceding sensation of weakness and depression—phenomena which most probably depend upon the augmentation of the disintegration of albumen just beginning, and the consumption of the static forces in store. The spasmodic contraction of the blood vessels of the skin, together with that of the smooth muscular fibers, causing the "goose skin," and even of voluntary muscles—causing the chattering of the teeth,—must give rise to a development of heat. In the beginning of febrile diseases, there is always a period to be found in which the morbid changes, the elevation of the interior temperature, the augmented secretion of urea and other changes in the urine, the increased frequency of the pulse and of respiration, and, finally, various subjective sufferings, show the existence of the febrile process, and with it the augmented formation of heat, even in the absence of the normal heat of the skin, or without an augmentation in its discharge; a period, therefore, in which an accumulation of heat may be presumed to take place.

The variations in the quantity of heat discharged, occurring during the fever, do not depend upon a total loss of the capacity of the skin to regulate the temperature of the body, but are rather owing to an abnormal excitability and irritation of the cutaneous vessels, brought about by the influence of the original cause of the fever, and probably reflected to the respective nerve-centers. It is the irregular and unequal contraction of these vessels, therefore, which prevent the equalization of the surplus of heat present. This irregularity of contraction and dilatation of these vessels during the febrile process has been satisfactorily proved by Senator and other investigators, who carefully observed it on the vessels of the ears of fevered rabbits and dogs; and it explains the alternate sensations of heat and cold, experienced by fever patients, especially before the fever is completely established.

From the above statements, concerning the elevation of temperature during the febrile process, it may be concluded that it is brought about by a disproportion between the abnormally augmented formation and discharge of heat; though the discharge at the height of the fever may be greater than normal, and at times even greater than the febrile formation of heat. The disproportion, therefore, is not equally prominent in each phase of the fever, and it is supposed that every hot stage has been preceded by a pyrogenetic, or cold stage, for the accumulation of the heat, just as, on the other hand, it terminates by a stage of defervescence with an augmented discharge of heat. In mild cases of fever, the same variations occur in the discharge of heat, only more gradually and less intense.

Of late years, numerous additional investigations, regarding the febrile process have been made, chiefly for the purpose of determining the exact relation existing between the central and peripheral temperature, and, also, of the relative temperature of different parts of the external surface of the body itself, as, for instance, that of the right or left side of the thorax during febrile diseases of the respiratory organs. In these investigations, the temperature of the axilla, or, in some instances, of the rectum, was taken as a standard of the internal heat, while the skin between the toes, or other parts of the surface were chosen for measuring the external temperature. But, though the results of these investigations, in general, corroborated the statements of Senator, and with it the neuro-pathological theory of Virchow, some contradictory statements have not been wanting. Thus, *Murri*,* basing his views upon the results of numerous experiments on animals, denied any dependence of the febrile process from the nervous system as chief regulator of the animal heat, and attempted to establish a bio-chemical hypothesis of fever in place of the neuro-pathological, now prevailing. He especially asserted that, in most cases of fever, no abnormal proportion between the peripheral and central temperature could be shown, and that the difference beween these temperatures were neither more variable, nor much greater than in the normal condition; but, on the contrary, often, and for a long time, remained unchanged during the course of the disease; frequently, even, the surface would be warmer than the central organs. *Jacobson*,† who experimented on a number of fever cases of pericarditis, pleuritis, typhoid fever, pneumonia, articular rheumatism, and tertiary intermittent fever, made use of a thermo-electrical apparatus. For the determination of the peripheral temperature, he introduced electrodes, in the form of fine needles, beneath the upper layer of the epidermis upon different parts of the cutaneous surface; for the central temperature the axilla was chosen. The results of these examinations were very inconstant. While, at one time, the difference of temperature between the axilla and the upper layers of the cutis was less during the fever than during the apyrexia, it would be greater at other times. Thus it varied irregularly between different places of the skin in the same individual, for, while the thermometer showed one and the same degree of temperature in the axilla, that of the skin was observed to undergo very considerable variations. The difference between the central and peripheral temperature was found to be in no way less than during the apyrexia. The same results were obtained in measuring the comparative temperature of the mouth and skin. These observations showed that at the acme of the hot stage, also, an alternate contraction and dilatation of the

* L. c. für das Jahr 1875, Vol. I, p. 281.

cutaneous blood-vessels takes place, and that during this stage, the quantity of blood in these vessels at the periphery of the body, and, in consequence, also the discharge of heat, are subject to variations within extended limits not only at different times, but simultaneously on different places. *Schuelein*,* with the view of answering the same question, instituted a series of thermometrical examinations on fever patients, for which he had availed himself of maximal thermometers with very small cylinders, corresponding very accurately with each other, and of which the one was introduced in the closed axilla, while the other was put between the first and second toe in such a manner as to completely embrace the bulb containing the mercury. The patients were quietly lying in bed under a light cover ; the temperature of the room was almost always the same, varying with some exceptions, between 18.0° and 20.0° C. In healthy individuals, it was found that while the temperature of the axilla remained nearly constant, that between the toes showed great variations. In the course of typhoid fever, peritonitis, acute articular rheumatism, erysipelas, endometritis, miliary tuberculosis, and cheesy pneumonia, continuous variations of temperature of the skin, not corresponding with those of the axilla, took place. In typhoid fever, they were observed within considerably wide limits, even when measured every quarter of an hour. During a severe frost a descent of the temperature of the skin nearly coincided with a rise of that in the axilla. Another series of observations, the results of which corroborated the statements of Jacobson and Schuelein, were made by *Schuck*.† *Wegscheider*,‡ who made his examinations on pneumonia and typhous patients, states, that the temperature of the periphery was subject to great variations, not running in any way parallel with those of the axilla. They were greater during the fever than in the feverless condition, a fact which he regards as depending upon an abnormal state of excitation of the cutaneous vessels.

A number of other observations have been made on this subject, which it is needless to cite, as they likewise corroborate the

† L. c. für das Jahr 1877, Vol. I, p. 219.

variations occurring in the contraction and dilatation of the cutaneous vessels during the febrile process.

In reviewing the preceding sketch of the febrile process, it will be found that though the chemical and physical processes concerned were satisfactorily explained, nothing was said which could throw any light upon the primary cause inducing the abnormal phenomena characteristic of fever. But as, in order to form a correct idea of the general pathology of yellow fever, it is important to have a clear understanding, not only of the chemical and physical processes concerned in the febrile process in general, but also of the principal views which at the present time are entertained as to its primary cause, a brief discussion on the subject is demanded.

Judging from what is known about the chemistry and physics of the febrile process, as demonstrated in the preceding sketch, the most prominent phenomenon of the whole is the over-production of heat, depending chiefly, but not entirely, upon an increased disintegration and exchange of matter. This heat, however, though its formation may from the beginning of the fever be going on at an equal rate, is not equally distributed throughout and discharged from the body. In consequence, the elevated temperature in fever, as once said before, is brought about by a disproportion between the abnormally augmented formation and the not in the same degree augmented discharge of heat, though the discharge at the height of the fever may be greater than normal, and at times even greater than the febrile formation of it. But, while the chief cause of the latter is the augmented disintegration and exchange of matter, the cause of the unequal discharge of heat, depending upon an abnormal and irregular contraction of the cutaneous blood-vessels, must be looked for in the nervous system. The question to be answered in relation to the primary cause of fever, therefore, is whether the increased waste of matter in the organism is the result of the accumulation of the normal quantity of heat within the body, depending upon the diminished discharge from the surface, on account of the irregular and abnormal contraction of the blood-vessels of the skin,—or whether the augmented disintegration of matter, especially the albumen, preceded the contraction of these vessels, and by some irritative influence upon the respective nerve-centers had perhaps, itself, been the cause of the contraction. In the one case, the process would have commenced in the nervous system, and be in concert with the neuro-pathological theory; in the other, its starting point would have been the blood, and it would correspond to the principles of humoral pathology. In examining the subject a little closer, it will be found that neither the one nor the other theory can be strictly and exclusively applied to every form of fever. For, in taking, for example, the febrile process as it accompanies the so-called infectious diseases which are supposed to depend upon a certain poisonous material entering the blood through the avenues of the lungs and the alimentary canal, it will be obvious that the process commences in the domains of humoral pathology; while, on the other hand, the febrile process following the shock of a severe bodily injury, and representing the so-called symptomatic fever, most probably took its start from the nervous system. Therefore, the primary cause of some fevers will be strictly humoral, whilst that of others will be nervous. But it must be remembered, that though in a number of fevers the effect of the cause, representing some noxious matter, is directly made upon the blood, it may also, through the medium of this liquid, very rapidly extend to the nervous system, which, reacting upon the one or other organ of the body, may induce serious pathological changes. And, in the reverse, in those fevers associated with severe surgical injuries, the disturbance and derangement in the nutrition of the injured parts may give rise to a certain pathological condition of the blood, which, through the medium of the nervous system, may conjure up the febrile process. We see, therefore, that the febrile process in general cannot be made exclusively to fit one or the other theory, but being of a complex nature, remains independent.

The neuro-pathological theory of *Virchow*, applied to the febrile process, explains many of the phenomena manifested, as, for instance, that of the irregular discharge of heat from the cutaneous surface by an irregular spasmodic contraction and dilatation of the cutaneous blood vessels, caused by a morbid irritation of the vaso-motor nerves. But, although the phenomenon of the non-equalization of heat is of a nervous origin, it does not necessarily follow that the augmented disintegration and exchange of matter is likewise the direct result of a perverted nervous action, for it has, as yet, not been positively shown that the original production of animal heat depends solely upon the nervous system, unless we presume the existence of special nerve-centers performing the function of regulating the exchange of matter, and with it the production of heat. The experiments of *Magendie*, *Bernard*, *Buettner*, *Rollet*, *Mantigazza*, *Schiff*, *Vulpian*, *Obolensky*, *Haidenhain* and *Legros*, made on animals in relation to this subject, and the clinical observations of *Samuel*, *Weir*, *Mitchell*, *Morehouse*, *Keen*, *Charcot*, *Erb*, and others, on man, however, seem to indicate the existence of so-called "trophic" nerve-centers, which regulate the nutrition of the tissues and organs, though on the other hand, a number of other investigators have obtained negative results from their experiments. The decrease of heat in paralyzed limbs, also, points to the influence of the nervous system upon the exchange of matter in the paralyzed parts. A number of experiments, consisting in the section of the spinal marrow, have likewise been made on animals by *Bezots*, *Ludwig*, *Thiry*, *Tscheschichin*, *Schiff*, and others, for the purpose of determining the variations of temperature in the extremities, with other observations on cases of severe injuries of the spinal marrow of man; these, however, may be passed over, as the results obtained are to a certain extent contradictory, and only seem to indicate local disturbances in the function of the blood vessels.

Now, even in recognizing the doctrine of trophic nerve-centers and nerves, and in admitting that the exchange of matter in the organism, constantly taking place in the fluids and solids, depend upon nervous stimuli, a special cause will still be required to act as such, and, accordingly, it may be presumed that in a similar manner as the nerve-centers presiding over the circulatory and respiratory functions, are stimulated to their rythmical reflex actions by the relative quantity of oxygen and carbonic acid in the blood, the normal combinations and decombinations of matter, constantly taking place in the blood and tissues, may form a stimulus for the trophic centers. As long as the organism remains undisturbed by external injuries, or by the influence of noxious substances, introduced into the blood from without, the trophic centers, then, will discharge their function in the same regular reflex manner as the respiratory; the exchanges of matter will take place in their just proportion, *i. e.*, each atom of matter will be replaced by an atom of fresh matter, and the normal nutrition of the tissue will be preserved. At the same time, the quantity of heat liberated by the exchange of matter will be counterbalanced by an equal discharge, regulated by the normal contraction and dilatation of the cutaneous vessels. But, as soon as any foreign substance, that is, a body incapable of being assimilated by the organism for the rejuvenation of the tissues, and therefore incapable of becoming a constituent of the latter, enters the blood, it will be removed by one or the other of the secretory organs, the only outlets from the organism. There are a large number of substances, some of which are used as medicines, which thus may enter and leave the organism without injury, and be detected in the secretion, while there are others, which, after entering, will, either catalytically, or by entering into combination with the organic constituents of the blood and tissues, exert a noxious or fatal influence upon it. To these belong the so-called infectious poisons with which we have to deal in this treatise.

In the symptomatic fevers, the morbid impression may be carried to the vaso-motor or trophic nerve-centers by the sensory nerves of the skin, or of the traumatic lesion, affecting them in the same manner as the respiratory centers are affected by sudden impressions made upon the skin, etc.; in the fever of infectious diseases, however, experience teaches that they are affected through the medium of the blood. Even Virchow, when founding the neuro-pathological theory, admitted this probability in saying:* "The rapidity with which febrile phenomena appear and disappear, likewise points to a cause residing in the nervous system, though the noxious material, affecting the nervous system, may be sought in the blood." And, further on: "In the study of the chemical nutritive processes of the body, it is of advantage to exclude the nerves as long as possible, as it has been shown that

* *Virchow.*—Handbuch der Speziellen Pathologie und Therapie, 1854, Vol. I, p. 36.

they exert no other influence, but in determining quantity or, acting as excitors or moderators, for all qualitative changes come from other directions."

In studying the general pathology of yellow fever, we must keep in mind that the clinical phenomena exhibited during its course, and the pathological changes met with after death, *do not*, as I have hinted at before, *exclusively* belong to this disease, but are equally observed, though in a more or less modified form, in other infectious, and even non-infectious diseases. It is only when they are considered as a totality, that they may represent the characteristic features of the disease. Thus, the continuous type of the febrile process witnessed in yellow fever, together with the congestion and parenchymatous infiltration and degeneration in various organs, are equally observed in other infectious diseases, particularly those of a contagious nature. The chief difference seems to lie in the specificity of the noxious poison, and in its tendency to affect particular organs in the different diseases, while its general effects upon the whole organism in these affections bear much resemblance to each other.

In the beginning of this treatise, I have already remarked that, in all cases of yellow fever, as well as of other infectious diseases, there is a prodromal stage, during which the poison exerts its noxious influence upon the blood, and primarily deranges the normal exchanges of matter. The symptoms of this stage, consisting, as we have seen, in a general discomfort, anorexia, lassitude, headache, etc., are decidedly nervous in character, from which fact it may be presumed that it is the nervous apparatus, which, first of all others, experiences the effects of the noxious poison. Now, although it is the blood with which the poison comes first into contact, it remains nevertheless difficult to determine whether it is directly carried by this fluid to the tissues of the nervous apparatus to make a direct impression upon them, or whether, in one way or other, it first contaminates the plasma of the blood, and, accordingly, makes an indirect impression upon the protoplasm of these tissues by deranging their normal nutrition. The latter supposition appears to be the most probable. At any rate, the influence which the poison at this period of the disease exerts upon the nervous system, is depressive in its nature, and appears to be directed to the vaso-motor centers. The immediate effect of a depression of these centers is a relaxation of the muscular elements in the walls of the blood vessels, especially of the smaller arteries, followed by a dilatation of these vessels and a hyperæmic condition of a moderate degree, upon which the prodromal phenomena seem to depend. Judging from the degree of the headache, it appears that the hyperæmia of the brain, upon which it depends, is at this period principally confined to the pia-mater, though the minute vessels of the cortex cerebri, also, may be slightly over-filled with blood. The sympathetic ganglia likewise suffer from the effects of the poison, and, moreover, exert their depressing influence upon the functions of the liver, stomach and kidneys. The degree of the prodromal symptoms in the different cases, is obviously proportionate to the quantity and intensity of the poison absorbed, but also to the particular susceptibility to the noxious influence of the poison by the person infected. In persons very susceptible to the absorption and influence of the poison, and who are exposed to it in its intense form by proximity to a severe case of yellow fever, the prodromal stage, therefore, may be comparatively short, while in others, not as susceptible, and exposed to the poison in a milder form, the prodromal phenomena may be milder, and extended over a longer period of time. From this difference, observed in the severity and length of the prodromal stage in different cases, we may presume that the abnormal disintegration and exchange of matter in the organism, resulting from the effects of the poison, must have proceeded to a certain extent, before the febrile process can start into action. The augmented quantity of urea, found in the urine before an elevation of the normal temperature of the body can be perceived, shows the correctness of this supposition. But, while the first impression of the poison upon the nervous system is of a depressive character, a sudden change takes place with the true commencement of the febrile process, evidently owing to a certain reactive effort of the vaso-motor nerve-centers, and manifesting itself in the abnormal excitability and irritation of the cutaneous blood vessels. It is impossible to decide whether this irritation proceeds from the vaso-motor nerves of these vessels, or from the sensory nerves of the skin, to be propagated to their respective nerve-centers, thus constituting a simple reflex action; or, whether it originates in the latter themselves, and is originally caused by the now concentrated effects of the infectious poison, or by the products arising from the augmented disintegration of albuminous substances, accumulated within the blood, and increasing the previously commenced disturbance of nutrition. The first effect of the augmented excitability of the vaso-motor nervous apparatus, then, is observed in the contraction of the cutaneous blood vessels, giving rise to the phenomena of the pyro-genetic stage, the mildness, severity, or duration of which evidently depends upon the quantity and intensity of the fever poison in the blood. Although those initiatory symptoms of the febrile process are subject to great variations in different cases of the disease, I do not think that they are ever entirely absent, but that, even in those cases in which no regular chill is observed, an alternate sensation of cold and heat, indicating the irritation of the respective nerve-centers, is experienced by the patient,—while in others, in which a regular chill, or even rigor, occurs, the spasmodic contraction of these blood vessels is more decidedly pronounced. As soon as the nervous energy, causing the contraction, is exhausted, a relaxation of the muscular fibers in the walls of the blood-vessels, followed by a dilatation, takes place, and in consequence of a larger quantity of blood coming from the central and warmer parts of the body, passing now through the vessels, the temperature of the skin gradually rises, until the hot stage of the fever is fully established.

In considering the disturbance of the nervous functions in yellow fever, we must not imagine that a depression or excitation of the nervous tissues takes place simultaneously throughout all parts of the nervous system, and that in consequence the blood vessels of all organs must be in a corresponding state of dilatation or contraction. On the contrary, as will be seen, the vessels of one part of the body may be relaxed, while those of another may have preserved their normal tonus, or, as in the case of the cutaneous vessels, may alternately be contracted or relaxed. In order to appreciate correctly the relative disturbances in the functions of the various organs of the body, and the pathological changes taking place in the blood and tissues during the whole course of an infectious disease like yellow fever, one should be familiar with all the anatomical and physiological minute details of the entire organism, the study of which, alas! is rather neglected by the majority of practicing physicians. Without the knowledge of these details, which, after all, constitute the basis and greater portion of true medical science, the physician can only form an indefinite, vague idea of the normal and pathological processes taking place in the human organism, while, with it, he may represent to his mind the entire mechanism of the body in all its details, and in full operation.

Already, before the commencement of the hot stage, pains in the joints and limbs, as we have seen, are added to those in the head, increasing in severity with the rise of the temperature, until the fever is fully established, when they either disappear, or continue in a milder form during the hot stage. These pains, which are most severe in the lumbar region and pelvis, must probably depend, as mentioned once before, upon the congested condition of the blood vessels of the pia mater, and of the spinal marrow in the lumbar region, whence they are reflected to the joints and muscles affected. Whether these pains, as well as those in the head, are simply owing to the pressure of the congested blood vessels upon the nervous substance, or whether their severity partially depends upon the direct noxious influence of the infectious poison upon the nervous tissue of the affected parts, I will not venture to decide, though the latter supposition appears probable from the fact, that, in fatal cases, these pains generally disappear before death, while the blood vessels are still found congested with blood corpuscles after death.

The nervous apparatus, through the influence of which the circulation of the blood is sustained and regulated, is quite complex, and consists of several nerve-centers. While the rythmical movements of the heart, by which the blood is kept in circulation, are most probably caused by the influence of certain nervous ganglia situated in the muscular substance of the heart itself, they are regulated by other centers situated in the medulla oblongata, and operating through the pneumogastric and sympathetic nerves; the nerves which the heart receives from the cardiac plexus are derived from these sources. The fibers of these nerves, however, differ very considerably in their function, for while the pneumogastric fibers transmit only inhibitory stimuli, the other transmit stimuli which accelerate the heart's action. Accordingly, an irritation of the fibers of the pneumogastric nerves will be followed by a retardation, or even arrest of the movements of this organ, while an irritation of the centers in the medulla oblongata will produce an acceleration, as long as their communication with the heart through the spinal marrow, rami communicantes, first thoracic ganglion, etc., is not interrupted. An acceleration of the heart's action will also be produced by an increased pressure of the blood within the cavities of the organ, as may be caused, for example, by a contraction of the smaller arteries, depending upon an irritation of the vaso-motor centers, likewise situated in the medulla oblongata. From this it is easy to understand how an irritation of the medulla oblongata, produced by the noxious influence of the yellow-fever poison, will give rise to one of the most prominent phenomena of the febrile process, the frequency of the pulse. Besides this, the elevation of temperature also, depending upon the augmented disintegration and exchange of matter, initiating the febrile process, forms another cause for the accelerated action of the heart. The latter assertion, however, must appear contradictory to the circumstances, before mentioned, of the falling of the pulse on the second day, a time when the temperature is still rising. But may not this phenomenon be owing to an irritation of the inhibitory centers of the pneumogastric nerves, set up at this time?

The abnormally augmented disintegration and exchanges of matter in the organism, originally caused by the presence of the infectious poison in the blood, and giving rise to an increased formation of urea and other products, as well as to an augmented quantity of heat, will now continue, until the poison is destroyed, or eliminated from the system, while, at the same time the products arising from these abnormal processes, or the direct action of the poison itself upon the nervous tissues, or upon the morphological elements of the blood, may give rise to the abnormal excitability and irritation of the involved nerve-centers with their nerves.

The morbid excitability of the nervous tissues, however, does not appear to be confined to the centers and nerves already mentioned, but, moreover, extends to other parts of the nervous system, giving rise to other phenomena of the hot stage, consisting in the congestion and deranged secretion of several organs. Thus, the congestion of the blood vessels of the conjunctiva very likely depends upon a neuro-paralysis of the vaso-motor nerves, and is similar in character, though inferior in degree, to that produced in the blood vessels of the rabbit's ear by the well known experiments of Claude Bernard of dividing the sympathetic nerve in the cervical region. The deeper seated pains of the eye, and also the slight photophobia, are phenomena which depend upon the irritation of the trigeminal nerves and their centers. In the same manner, the congestion and irritation of the mucous membrane of the pharynx, œsophagus, and stomach are explained; for in considering the fact that all the minute blood vessels of the brain, but particularly those of the pons varolii, corpora quadrigemina, and medulla oblongata, are found after death in a congested condition, completely filled with blood corpuscles, there will be no difficulty in understanding the congestion and irritation of these mucous membranes, if we remember that the nuclei, or centers, of the trigeminal, glosso-pharyngeal and pneumogastric nerves, supplying these parts, are situated near each other, alongside of the aqueduct of Sylvius and the floor of the fourth ventricle of the brain, and that all these nerves closely communicate with the sympathetic. Judging from the scarlet-red color of the congested vessels of the conjunctiva, gums and edges of the tongue, during the first part of the hot stage, the congestion is arterial in its nature, that is, depending upon a relaxation of the arterioles and the ensuing afflux of blood. As regards the stomach, it hardly needs mentioning that the nausea and inclination to vomiting are due to the irritation of its mucous membrane, the secretory function of which, however, is not suspended, but only deranged, as shown by the quantity of frothy mucoid liquid thrown up at this time. As regards the liver, kidneys and supra-renal bodies, I have already mentioned, when discussing the pathological anatomy of these organs, that the parenchymatous degenerations, observed after death, have been initiated by a congestion of the blood vessels, depending most probably upon the same causes as that of the mucous membranes above discussed. But, before passing to the discussion of these parenchymatous changes, I must make some remarks on the condition of the blood.

In the section of this treatise devoted to the pathological anatomy of the blood and a number of organs, I showed that, besides an unusual tendency of assuming the mulberry or thorn-apple forms, manifested by the colored blood corpuscles, nothing abnormal, or foreign, could be detected in this fluid, when carefully examined directly after it had been removed from the living patient; while, on the other hand, in almost every organ in which a congestion had occurred, numerous extravasations or infiltrations of the coloring matter of the blood corpuscles, the hæmo globin, into the parenchyma were met with. The question arises, therefore, whether the hæmoglobin parted from the colored blood corpuscles only in those places where the extravasations occurred, by virtue of the existing retardation of the circulation in the congested capillary vessels, or whether it escaped from the corpuscles while they were still in an active circulation. In answering this question, it may be said, that the direct cause of these extravasations evidently was the retarded circulation, but that there also existed a remote cause, consisting in a want of coherence between the hæmoglobin and the protoplasm of the blood corpuscles, facilitating, as soon as the retardation took place, the escape of the former from the latter. And, it may further be presumed, that not all the hæmoglobin escaping from the blood corpuscles, passed through the walls of the vessels, but that perhaps the greater portion became mixed with the liquor sanguinis and was carried into the general circulation, giving rise to the jaundice. Thus, it will be seen, *that the icterus in yellow fever is not owing to the presence of bile in the blood*, as is believed by a large number of physicians, *but to the presence of free hœmoglobin*, and represents in truth the so-called "*hematogenous*" jaundice. A "*hepatogenous*" jaundice cannot take place, as the larger as well as the smaller hepatic ducts are found perfectly open, and as the secretion of bile during the disease, in the majority of cases, is rather diminished, or even suspended.

As regards the presence of free hæmoglobin in the blood, however, some doubt might still be expressed on account of its not having been demonstrated by the microscopical examination of so many specimens of fresh blood, taken from the living patients. The reason for this failure is, that the quantity of hæmoglobin contained in the exceedingly thin layer of blood required by microscopical examination, is too small to exhibit its yellow color to the eye of the observer, and that in order to be seen, the blood should be saturated with the coloring material to such a degree as would require a quantity larger than would be consistent with life. Free hæmoglobin, therefore, may be present in the circulating blood without being detected by the microscope. The faintly yellowish tint, appearing, in a number of cases, as early as the third day of the disease, in the conjunctiva and the skin, corroborates this assertion ; though, in the majority of cases, the jaundice appears later, if it appears at all during life. The yellowish tint of the conjunctiva and skin, however, is not produced by the free hæmoglobin still contained in the circulating blood, as its presence is not perceived until it has escaped from this fluid into the juices of the surrounding tissues, to be absorbed by the cells of the epithelium of the conjunctiva, or those forming the lowermost stratum of the epidermis of the skin.

The extravasations of hæmoglobin into the parenchyma of various organs, which I have observed to occur in yellow fever, fully corroborate the generally accepted fact mentioned before, that in the febrile process, it is these nitrogenous tissues, rich in potassa and hæmoglobin, especially the colored blood corpuscles and muscles, which are most prone to disintegration, and which first suffer from the effect of the poison.

Let us now direct our attention to the liver. Although in most cases of yellow fever, as I have shown, the traces of congestion of the blood vessels, observed after death in this organ, are not as conspicuous as in other organs, there remains for the reason already given, no doubt but that the liver is one of the first organs which experience the deleterious effects of the poison, and participates in the general hyperæmia resulting from the depression of the vaso-motor nerves, manifested during the prodromal stage. But, like in the stomach, as soon as reaction takes place, the secretion of bile is resumed, and, very likely, for a short time abormally increased, as may be inferred from the vomiting of bilious matters, observed in a number of cases during the commencement of the disease. Judging from the fact that most cases of miasmatic fevers, even the simple intermittent, are accompanied by some derangement of the biliary functions, I have always been inclined to regard the liver as that organ through which the organism makes the first attempt at eliminating the miasmatic poison by stimulating its secretory function. The same may take place in yellow fever, as above mentioned, though the abnormally stimulated secretory function will soon be exhausted, and an opposite condition prevail. But, besides this stimulation through the secretory nerves, it appears very probable that the liver receives another stimulus, proceeding from the part of the blood, for the purpose of removing, and converting into bilirubin, the free hæmoglobin escaped from the colored blood corpuscles. But like even in the normal condition, the amount of labor which the parenchyma of a gland can perform, stands in a certain proportion to the quantity or number of its secreting cells, only a small portion of the free hæmoglobin can be converted into bilirubin by the liver, while the rest extravasates through the capillaries of the different organs and tissues as we have seen, to be absorbed by the surrounding cells, or to mingle with the fluids of the tissues. Thus, the abnormal stimulation of this organ, accompanied by the increasing disturbances of the nutrition of the whole organism, soon leads to a state of exhaustion.

But, while these functional changes are taking place in the liver, the abnormal processes in the blood, relating to the augmented disintegration of the albuminous constitutents, and their metamorphosis into the final products urea, carbonic acid and water, are likewise running their course. The albumen, thus undergoing disintegration, may be derived from two sources, *i. e.*, from the circulating albumen of the blood, designated by *Voit* the "store albumen," or from the albumen of the tissues, the "stable" or "tissue-albumen." As the former, on account of the small quantity, is soon consumed, it is especially the latter which suffers the greatest losses during the febrile process. In consequence of the augmented formation of urea, consuming considerably more oxygen than is normal, the other final products, requiring still more oxygen for their formation, must fall short in quantity, and the combustion of the non-nitrogenous constituents be retarded. But, as an abnormal quantity of albumen is decomposed without a corresponding quantity of non-nitrogenous material, such as fat, the latter accumulates in the blood, and gives rise to the *fatty infiltration* of the different organs, already described.

Fatty infiltration of the liver, besides being met with in infectious diseases, also frequently occurs in chronic diseases accompanied by great exhaustion of the system, such as phthisis, chronic diarrhœa, dysentery, etc., in which the normal processes of nutrition and assimilation are severely deranged, so that a part of the albumen, destined for the rejuvenation of the tissues, instead of being appropriated for this purpose, becomes converted into fat; the same occurs through the imperfect combustion of the non-nitrogenous substances in the lungs. Generally, in these diseases, the nutrition becomes deranged only gradually, and, if fatty infiltration of the liver takes place, the process advances, in most instances, at the same slow rate of the original disease. In yellow fever, however, the case presents a different feature. Through the deleterious influence of the infectious poison, the disintegration of albumen and the exchanges of matter take place quite rapidly, and the fatty infiltration of the liver may commence at a comparatively early period of the disease, and, in advancing, seriously interferes with the neighboring organs. For, besides the diminution and final suspension of the biliary secretion which it causes, it also interferes with the portal circulation by the pressure, which the abnormal amount of fat, deposited by the blood and absorbed by the hepatic cells, exerts upon the capillaries and inter-lobular vessels. And it is this pressure by which the congestion, representing the first stage of the pathological process in this organ, is gradually reduced. In advanced cases of fatty infiltration, therefore, the minute vessels are very rarely found over-filled with blood; on the contrary, the capillaries, as we have seen, are mostly found empty. The disturbance in the portal circulation, however, will soon be felt by the tributaries of the portal vein, for, while the passage of the blood through the liver is rendered more difficult, a congestion of these veins will be the result, which, in extending backward to the minute venules of the mucous membrane of the stomach and intestines, will give rise to that peculiar congestion of these organs, already described. In the stomach, this congestion is mostly accompanied by an infiltration of hæmoglobin into the surrounding tissues; and, in severe cases, ruptures of the minute venules, or capillaries, situated between the epithelial and glandular layers, will occur, and give rise to that much dreaded clinical phenomenon, known as "black vomit."

The vomiting of black matters in yellow fever has always been regarded as a most unfavorable symptom. The morphological elements of the blood which these matters contain are derived, as we have seen, from the minute hæmorrhages caused by the rupture of the vessels just mentioned. It is quite obvious that such a hæmorrhage must result in the direct relief of these minute vessels from their congested condition, and that the small quantity of blood, thus lost, is in itself inadequate to cause the fatal issue of the case, though *it most certainly indicates a very diseased condition of the liver, as well as a low state of the whole organism;* and it is, in this respect, that it becomes an important factor in the prognosis of the case. Besides this, although the primary congestion and irritation of the stomach is, during the first three days, rarely accompanied by much pain, when pressure is made upon the epigastrium,—it will finally, when aggravated by the advancing fatty infiltration of the liver in the manner described, give rise to a sensation of dull pain and fullness in the organ, rendering pressure insupportable to the patient. The condition of the liver, and with it that of the whole organism, therefore, may be accurately diagnosed by that of the stomach, and will notify the physician of the approaching danger. Very rarely black vomit occurs before the fourth, or even fifth day; in most cases, perhaps, it takes place still later, at a time when the pathological changes in the various organs have attained a high degree, and when the nervous energy of the organism is nearly wasted, producing an almost complete depression and exhaustion of the patient.

If, after the elimination of the poison from the system, indicated by the cessation of the febrile process, the pathological changes of the parenchyma of the liver have not advanced to a very great extent; that is, if the protoplasm of the hepatic cells itself, has not undergone *true fatty degeneration*, and, if sufficient energy is left in the nervous organs to supply the wants of the organism—*black vomit may occur without proving fatal.* For, after the removal of the disturbing cause, the poison, the condition of the blood will gradually attain again its normal standard, the tissues will again be properly nourished, and, in consequence, the deposition of fatty matters into the parenchyma of the liver will cease, and, finally, the fat in the hepatic cells be reabsorbed by the blood. In this manner, quite a number of patients recover from black vomit. In fact, in most cases in which black vomit occurs, the patient feels relieved after the ejection of these matters, and imagines that he will get well. In children, especially, the occurrence of black vomit is not as often followed by a fatal issue as in adults. This is owing to the exchanges of matter, especially in those organs pertaining to organic life, being more active than in adult life; while, on the other hand, the organs of animal life, particularly those of the nervous system, are in children more impressible and sensitive.

In many fatal cases of yellow fever, especially those in which the third stage is protracted, the pathological changes of the liver do not remain confined to a simple *fatty infiltration*, but advance to a *true fatty degeneration* of the protoplasm of the hepatic cells, as I have described in the section of pathological anatomy. These cases, of course, as may be expected, prove necessarily fatal.

The relationship existing between the stomach and the liver, also exists between the latter and the intestines, and it is therefore natural that the same pathological phenomena should be observed in these organs, though inferior in degree. Accordingly, in a number of cases, hæmorrhages, similar to black vomit, are observed to take place from the mucous membrane of the small intestines, which, mingling with the mucous matters, pass per anum, though, sometimes, the black matters from the stomach may also be voided in this direction.

The infiltration and degeneration of the liver, associated with congestion and minute hæmorrhages of the stomach—black vomit—are so constantly met with in every fatal case of yellow fever, that they may safely be regarded as a characteristic phenomenon of this disease. In cases in which the black matters are not ejected during life, they will, almost always, be found in the system after death.

In taking now a glance at the kidneys, the pathological changes of which have already been fully discussed in their appropriate place, we find that these changes have, as in those organs already discussed, been preceded by a hyperæmic condition primarily depending upon the same cause, that is, a partial paralysis, or paresis, of the vaso-motor nerves. One of the first phenomena, depending directly upon the relaxed condition of the minute vessels, especially the arterioles, is the albuminuria, making its appearance quite early, on the second or third day of the disease. It is now generally accepted that this phenomenon, very frequently accompanying even mere functional disorders of the kidneys, chiefly depends upon an increased pressure of the blood upon the inner surface of the walls of the minute vessels of the glomeruli, as has already been noted. In yellow fever, the conditions necessary to the production of albuminuria, are rendered especially favorable by the relaxed state of the smaller arteries, so that, soon after the commencement of the febrile process, when the heart's action is increased, albumen may make its appearance in the urine, without being accompanied by other products indicative of organic changes in the organ. The degenerative processes commence at a later period, during the second stage of the disease, when the increased disintegration of albumen and exchange of matter, entailing a complete derangement of the nutrition of the various organs, has reached a higher degree. As in the liver, a slight reaction, resulting in a temporary stimulation of the secretory functions and following the general nervous depression, also takes place most probably in the kidneys. This stimulation may be caused by the accumulation of urea, or its components, in the blood, calling upon the kidneys for elimination from the organism. And it seems to be not impossible that this same cause, together with the deranged nutrition of the epithelial cells, lining the uriniferous tubules, may also give rise to that abnormal product of secretion of which the cylindrical infarctions are formed. The nature of this abnormal secretion. and the manner in which the different kinds of cylinders are formed, have already been so thoroughly discussed as to require no further remarks in this place.

It has already been mentioned that the degenerative changes in the parenchyma of the kidneys are not observed to have taken place in the same degree in all fatal cases of yellow fever, but, on the contrary, differ widely from each other. From this fact, it may be presumed, that in those cases in which these changes have been limited, death cannot be assigned to them, even if the urinary secretion should have been suspended for an abnormal length of time before this event took place. Even in those cases in which these changes have progressed to a considerable extent, there is a sufficient number of uriniferous tubules left open for the passage of the secreted urine. And if, in some cases, a total suppression of urine really takes place, it is hardly due to the obstruction caused by the infarctions, but much more probably by the general *atrophy* and *degeneration* of the epithelium. But, even if this were the case, the question whether the suppression of urine is the immediate cause of death, as is believed by a large number of physicians, remains still undecided.

The question whether or not the nervous symptoms accompanying certain organic changes in the parenchyma of the kidneys, and called "uræmic," really depend upon an accumulation of urea in the blood, has been agitated for a considerable number of years, and is as yet not definitely settled, though, in the course of this time several theories, in a modified form, have been advanced. Thus, while a number of physicians regard the accumulated urea in the blood as the immediate cause of uræmia, others accept the theory of *Frerichs*, according to which these nervous phenomena are owing to the carbonate of ammonia resulting from the decomposition of the urea in the blood; while others, again, attribute them to the presence of the extractive matters. *Traube*, even, rejected these theories altogether, and attributed the symptoms of uræmia, consisting in convulsions, coma, vertigo, etc., to œdema of the brain. Since these different theories were advanced, a considerable number of experiments and observations have been made in relation to the subject, for the purpose of ascertaining the truth, and almost all the results obtained rather prove the fallacy of the uræmic theory. I shall cite the views of some of these investigators. Thus, *Chalvet** found during the uræmic attack a diminution of the urea in the urine and blood, rising again after the attack, but without attaining the normal quantity. Accordingly, he rejected the theory of Wilson and Frerichs, based upon a retention of urea in the blood, and rather regarded the retention of excremental matters in general as the cause of the uræmic phenomena. He, moreover, doubted that urea was formed in the tissues at all, but were rather a secretory product of the kidneys. *Budde*,† who made quite extensive observations on this subject, opposes the uræmic theory based upon the poisoning by urea, and particularly points to the fact, that a diminution of the secretion of urea before and during the uræmic attack does not constantly occur. He says that it is true that such a diminution takes place with a simultaneous decrease of diuresis, though the latter is in many cases rather abundant during the uræmic state. Four cases, which the author himself closely observed and described, showed an abundant diuresis, and three of them, besides, an excretion of urea in a comparatively large quantity, namely: 25.7, 27.6 and 22 grms. In 25 severe and fatal cases of uræmia, which he collected, there were five with abundant diuresis up to the commencement of the attack, in one of which the diuresis even rose to from 1200–2000 Ccm. during the attack. The conclusion drawn from this is, that, even if the excretion of urea is considerably diminished, or temporarily ceases altogether, uræmia must not necessarily follow; it rarely occurs in amyloid degeneration of the kidneys, neither in a cachexia, as leucæmia, notwithstanding the very scanty excretion of urine in these cases. Finally, uræmia does not necessarily follow upon long persisting anuria, accompanying certain diseases of the kidney and urinary passages. Budde observed himself a case of anuria lasting 40 hours, not followed

---

* *Virchow u Hirsch*—Jahresbericht f. d. Jahr, 1867, Vol. I, p. 336, and 1868, Vol. I. p. 226.

† L. c.—f. d. Jahr, 1874, Vol. I, p. 347.

by uræmia;* he furthermore reports a case of a woman, of 24 years, with highly albuminous and abundant urine—during several ca. 1700 Ccm., a few times even to 2300 Ccm.—and considerable œdema, in whom slight uræmic symptoms were observed, disappearing again with the decrease of the œdema. Two weeks previous to death, severe diarrhœa and vomiting, accompanied by scanty urine and disappearance of the œdema, occurred; and in the last 132 hours total anuria with no discharge per os or per anum, and without any uræmic symptoms. The autopsy revealed: chronic nephritis in the second stage, amyloid degeneration of the spleen, bronchiectasis and phthisis cavernosus pulmonalis. The author then shows the untenability of the theory, according to which uræmia is caused by a poisoning through the extractive matters, or through the carbonate of ammonia, and endeavors to prove the incorrectness of the assertion of *Jaccoud*, that toxic uræmia clinically differs from that depending upon anæmia and œdema of the brain, and that the former can be recognized by an œdema otherwise wanting, as he found in his 25 cases of fatal uræmia six with œdema and anæmia of the brain without œdema in other localities, and six others with the same condition of the brain, and inconsiderable œdema in other places. For this reason, Budde sanctions the theory of *Traube*, which assigns œdema and anæmia of the brain as the cause of uræmic phenomena. In order to show how frequent these changes in the brain are met with, he collected and tabled these 25 cases of fatal uræmia which he observed in the Communal Hospital of Copenhagen. In 18 of these cases, anæmia and œdema of the brain were found; in four others, accumulations of liquid in the ventricles and in the subarachnoid spaces of the brain were present; in two more there was anæmia alone, and in one only, the brain was normal. These statistics support the theory of Traube in a high degree.

More recent experiments, consisting in the injection of urea into the blood, have neither been followed by convulsions, but

* A remarkable case of suppression of urine, observed by my friends, Drs. D. W. Brickell and J. D. Bruns, occurred very recently in this city. It was a boy, nine years old, who suffered from congestive bilious remittent fever, with a complete suppression of urine—duly ascertained by the use of the catheter—for 67 hours previous to death. His intelligence was perfect up to the instant of death, and no trace of uræmia was observed.

rather by a rapid excretion of the urea, proving what has been said before.

From the observations, cited above, together with others that occurred in this city, we have reason to judge that in those rare cases of yellow fever, in which a total suppression of urine really takes place, we are not justified in assigning it as the true cause of the convulsions, occasionally observed before death. A suppression of urine, therefore, can only signify an extensive degeneration of the secretory structure of the kidneys, the epithelium, and an exhausted condition of the nervous system, similar to the black vomit, indicating the desperate condition of the liver. At the same time, it must be remembered, that the secretory function of the kidneys is really not totally suppressed in all cases in which suppression is reported, but that, if they were properly examined, many of them would turn out to be only cases of retention, while in others the urinary function would be found to be only temporarily suspended. Thus it has frequently occurred that in cases in which the physician had even based his judgment upon the results obtained from the use of the catheter when pronouncing suppression, the secretion of urine had only been suspended, and was resumed soon afterward.* In many cases, however, suppression of urine is pronounced without the catheter ever having been resorted to. The fact that urine is found after death in the bladder in, at least, half the cases of autopsies, corroborates this assertion. We may then reasonably suppose, that though total suppression of the urine may take place in exceptional cases, it is not the immediate cause of convulsions and death, but that, on the contrary, the latter are owing to the diseased condition of the brain.

In the preceding section of this treatise I have shown, that in the numerous fatal cases of yellow fever in which I examined the brain, not only the smaller blood-vessels of the pia mater but also those of the brain substance itself, were found congested with blood, and that, in almost all of these cases, the congestion extended throughout the whole organ, and particularly through-

---

* *Dr. J. Dickson Bruns* reported to me a case, which he observed during the epidemic of 1873, and in which the secretion of urine was thus suspended for 36 hours, at the expiration of which time it was fully resumed.

out the pons varolii and medulla oblongata, in which the vaso-motor, trophic and secretory nerve-centers are situated. The hyperæmia of the brain depends upon the same cause as that of other organs, that is, upon a depression of the vaso-motor nerves, followed by a relaxation of the arterial walls. Gradually commencing before the beginning of the fever, it manifests itself already in the headache and general nervous depression during the preliminary or initial stage of the disease, but increases with the appearance of the fever, when the action of the heart becomes more violent, and when the arteries, in consequence of the relaxed condition of their walls, are no more able to withstand the increased pressure of the blood. And as these vessels cannot recover their normal tonicity as long as the battle between the organism and the infectious poison is raging, the congestion of the brain becomes persistent, and increases the already existing disturbance of the nutrition of the nervous tissues of this organ. In consequence, a considerable amount of the nervous energy, which in the normal condition is destined to supply the various organs, is now lost by being converted into heat, a condition which will last until the original cause of these abnormal processes, the noxious poison, is eliminated from the organism. If this condition of things lasts only a limited time, no organic changes will take place in the nervous tissues, but, on the contrary, they will, with the subsidence of the fever and the accompanying exchanges of matter, regain their integrity, as happens in all cases of recovery from yellow fever, and also in most other cases of hyperæmia of the brain. In the more violent cases of yellow fever, however, in which the febrile process is prolonged and particularly stormy in character, the hyperæmia becomes persistent and manifests itself by violent delirium, convulsions, or other serious nervous phenomena, which have been regarded as depending upon the degenerated condition of the renal parenchyma, and the ensuing uræmia. That this is not the case, but that, according to the theory of Traube, they in reality depend upon an œdema, or any other diseased condition of the brain, deranging the normal functions of this organ, I have already sufficiently shown in the preceding pages, and can, moreover, corroborate by the œdema and anæmia of the brain which I observed, two years ago, in a man who had been for a number of years an habitual opium-eater, and who died under severe convulsions, to which he had already been subject before his death. In a number of fatal cases of yellow fever, the brain, as I have shown, is found œdematous, which here, however, depends upon a hyperæmia, a condition, it is true, opposite to that which Traube assigns to the uræmic convulsions. This apparent contradiction, however, is easily explained by the fact that some of the accompanying phenomena of anæmia of the brain, such as convulsions, may likewise be produced by a hyperæmia of that organ. In both cases, the symptoms depend upon a disturbance of the normal nutrition of the organ, arising, in the one, from a diminution, and in the other from a derangement, or complete interruption, of this function.

The deranged nutrition of the nervous tissues, caused by a persisting hyperæmia, will also eventually give rise to degenerative changes of these tissues, or their minute blood vessels. Such changes may be observed, in a number of fatal cases, in the fatty degeneration of the minute blood vessels and of ganglion-cells of the sympathetic ganglia, and, even, to some extent, in those of the cortex cerebri. The degenerations which I observed in the semi-lunar and first thoracic ganglia, consisting in the disappearance of the nuclei of the ganglion cells, and in the fatty and atrophied appearance which the latter themselves presented, cannot but exert a most deleterious influence upon the organs which they supply, by greatly diminishing the nervous energy which they need for the preservation of their integrity. With the degeneration of the nuclei of the ganglion-cells, which I regard as special reservoirs, or stores, of potential energy, the influence which these nerve centers exert upon the nutrition of their respective organs, must become considerably diminished. The pathological changes in the kidneys, supra-renal bodies, and liver, therefore, may stand in a close relationship with those observed in the semi-lunar ganglion, and other ganglia of the solar plexus. In the same manner may the changes in the muscular tissue of the heart depend upon those in the ganglion stellatum. But, judging from these changes, found in those sympathetic ganglia to which my examinations extended, viz., the ganglion stellatum, ganglion semilunare, and some others of the solar plexus, we may well presume that they had equally taken place in a number of other sympathetic ganglia, especially those of the cardiac plexus; and if, furthermore considering that the nerve centers which regulate the action of the heart, as well as its nutrition, send their stimuli through the ganglion stellatum and cardiac plexus, it becomes obvious that these changes cannot but exert a depressive influence upon the action and nutrition of this organ.

Thus it happens that, while during the febrile stage of the disease the heart's action is accelerated by the irritative influence of the poison upon these centers and ganglia, it is depressed during the second, but particularly the third stage, when the nervous energy becomes abnormally diminished by these pathological changes and the ensuing exhaustion of the nerve centers. And if this nervous depression passes beyond certain limits, a collapse of the organism must evidently be the result; but it may also terminate in paralysis of the heart, causing the death of the patient. In very severe cases, the nervous depression is frequently associated with a degeneration of the muscular tissue of the heart, which is likewise cutting off every chance for recovery. Excessive nervous depression, or paresis of the heart is, therefore, one of the most dangerous symptoms in the whole course of the disease, especially when it appears in the last stage of the disease, as it may then be associated with the degeneration of the muscular element, and, if not soon relieved by stimulation, must evidently lead to collapse. The feebleness and rapidity of the pulse, together with the diminution of the peripheral heat, especially of the extremities, indicate the approaching danger, the action of the organ being too low to properly circulate the blood through the peripheral vessels. The muddy, cyanosed appearance of the face, shortly before death, also depends upon this cause.

In taking a final review of the nervous phenomena of yellow fever, we find then that, while in the commencement of the disease they depend upon a paresis of the vaso-motor nerves, this condition is soon followed by an irritation of the different nerve centers mentioned, situated in the medulla oblongata, and giving rise to the temporary acceleration of the heart and the irregular contractions of the cutaneous blood vessels during the hot stage; but that, in the second and third stage, this abnormal excitation is relieved by a general depression and exhaustion of the nervous system, in many cases associated with paresis, or even paralysis of the heart. But though the latter condition, which mostly takes place in protracted cases, may be the immediate cause of death, the hyperæmia of the brain is not relieved, but persists in a more chronic form until the fatal issue, when its presence will be revealed by the autopsy. That in fatal cases, without regard to the immediate cause of death, the neuro-paralytic condition of the blood vessels persists until this event takes place, is corroborated by the larger cerebral arteries, even the basilar, being found filled with blood, which is not the case in other diseases in which death occurs from other causes and without hyperæmia of the brain, where the larger and smaller arteries contract during the agony, driving the blood through the capillaries into the veins.

The pathological changes, observed in the supra-renal bodies depend upon the same causes, and take place in the same manner as those in the kidney, liver, and other organs; but, for the reason that so little is known concerning the true function of these organs, I shall forbear making any remarks as to the part they may play in yellow fever.

In comparing the clinical phenomena and pathological changes of yellow fever with those of other infectious diseases, we find that they resemble each other in many points. Thus, in the typhoid diseases, such as typhus, typhoid, bilious typhoid, and relapsing fever especially, the same nervous disturbances, caused by the influence of the infectious poison and the ensuing derangement in the nutrition of the nervous tissues, are equally met with. The extravasations of hæmoglobin and the general tendency to capillary hæmorrhage, as well as other degenerative processes in the glandular organs are also observed in numerous cases of these diseases. But, notwithstanding the similarity existing in the above phenomena, there are some other important points, in which yellow fever essentially differs from these typhoid or other kindred diseases. The difference particularly concerns the socalled "blood-making" organs, the spleen and the lymphatic glands, which in the above diseases are almost always more or less affected, while in yellow fever they remain perfectly normal. This circumstance also explains the fact, that in these infectious diseases the blood is frequently found to have lost its coagulability, that is, it has in the true sense of the word become contaminated, or prone to decomposition, which never, or, at least, *very rarely* takes place in yellow fever, though it is believed by a large number of physicians. In the latter, the infectious poison appears to exert its noxious influence especially upon the morphological constituents of the blood, the colored blood corpuscles, inducing them to part with their coloring matter, the agent in the processes of oxydation taking place in the organism; and the congestions occurring depend less upon a true contamination of the blood, than, as has been demonstrated, upon a paresis or paralysis of the vaso-motor nerves.

Another difference may be found in the duration of the febrile process, the extent of which seems to be typical, as regards its length of time, to each individual infectious disease.

Besides, many of the phenomena of yellow fever are also met with in those infectious diseases, known as "*exanthemata*," and in the different forms of miasmatic disease, so that only a few symptoms remain to be regarded as *pathognomonic* of this disease. This leads us to the question, How then, may yellow fever be distinguished from the above named diseases, and how may sporadic cases be correctly diagnosed during the absence of an epidemic?—questions quite difficult to answer. In the commencement of the disease, and, even, after the febrile process has started into action, it is almost impossible to make an infallible diagnosis, as a number of other infectious diseases commence in the same manner, accompanied by the same phenomena. In the diagnosis of yellow fever, therefore, *the whole complex of symptoms must be taken into consideration.* On the second day, the general observation concerning the relative state of the pulse and temperature, to which I have referred before, may be applied. This observation consists, as will be remembered, *in the falling of the pulse on the second day, with a simultaneous rise of the temperature of the body.* The presence of albumen in the urine on the third day, also, may to a certain extent serve as a pathognomonic symptom. If the disease runs on to its third stage, and black vomit should appear, all doubts about the nature of the disease, of course, must vanish. In fatal cases, the autopsy must decide the question from the pathological changes met with in the different organs, for *fatty infiltration of the liver, associated with that peculiar congestion of the stomach, together with black vomit*, are pathognomonic phenomena of yellow fever.

As regards the prognosis of the disease, the reader may draw his own conclusions from what has been said in the preceding part of this treatise.

[TO BE CONTINUED.]

